# Adopting the rapamycin trapping assay to track the trafficking of murine MHC class I alleles, H-2K^b^

**DOI:** 10.1186/s12860-015-0077-1

**Published:** 2015-12-29

**Authors:** Esther Ghanem, Mohammed Al-Balushi

**Affiliations:** Department of Biology, Faculty of Natural and Applied Sciences, Notre Dame University, 72, Zouk Mosbeh, Keserwan district, Lebanon; Department of Microbiology and Immunology, Sultan Qaboos University, Muscat, Oman

**Keywords:** (QC) quality control, (MHC) Major histocompatibility complex, Intracellular trafficking, Protein sorting, Rapamycin trapping assay

## Abstract

**Background:**

In mammalian cells, the quality control (QC) of properly folded proteins is monitored in the early secretory pathway, particularly in the endoplasmic reticulum (ER). Several proteins, including our protein of interest, major histocompatibility complex class I (MHC class I), can bypass the first line of ER-QC and reside in post-ER compartments in an unfolded form. Such forms entail both monomeric and dimeric structures that are devoid of peptides and thus cannot fulfill the immunological function of antigen presentation at the cell surface. MHC class I structures become mature and properly folded once loaded with the appropriate peptides in the framework of the peptide loading complex (PLC). Despite the flood of information on the diverse trafficking behavior of different MHC class I alleles, there is still controversy on the actual trajectory followed by improperly folded murine MHC class I alleles, namely H-2Kb. In this study, we employ an *in vitro* rapamycin trapping assay, live cell imaging, and a biochemical COPII budding approach to further investigate the trafficking of H-2Kb beyond the level of the ER.

**Results:**

We confirm the egress of H-2Kb in an unfolded form to a post-ER compartment from where they can cycle back to the ER. Deciphering the exact identity of the post-ER compartment by laser scanning microscopy did not only point to the existence of the ERGIC and cis-Golgi compartments as residency areas for unfolded proteins, but also to the involvement of an addional compartment, that lies in close proximity and possesses high resemblance to the aforementioned compartments. Interestingly, we were capable of showing using the same rapamycin trapping assay that H-2Kb can undergo a potential maturation event during their cycling; this is attained upon addition of peptides and trapping of accumulated post-ER molecules at the cell surface.

**Conclusions:**

Our findings deepen the understanding of H-2Kb trafficking outside the ER and pave the way to decipher the role and the trafficking of certain PLC chaperones, such as tapasin, throughout H-2K^b^ post-ER QC. Finally, we demonstrate the plausible usage of the rapamycin assay to assess the trafficking of defected proteins especially in diseases and under therapeutic studies.

## Background

Like other transmembrane glycoproteins, major histocompatibility complex (MHC)^1^ class I molecules are subject to cellular quality-control (QC) during folding and maturation. A substantial body of work has investigated the intracellular trafficking of MHC class I from its biosynthesis in the endoplasmic reticulum (ER) to its deposition at the cell surface for its proper function. To initiate the journey, the free heavy chain (FHC) of MHC class I interacts with the ER chaperone protein calnexin and with the protein disulfide isomerase ERp57 until it has folded and associated with the light chain, beta 2 microglobulin (β_2_m). In case they pass the initial folding hurdle, they can bind to the light chain, β_2_m, forming dimers that are recognized by the lectin chaperone, calreticulin; together with three other proteins, tapasin, ERp57, and TAP, they form the class I peptide loading complex (PLC) [1]. Tapasin plays a crucial role in the maturation of MHC class I molecules by editing high affinity peptides onto MHC class I grooves. Tapasin also bridges MHC class I to TAP, the transporter associated with antigen processing, thereby enhancing peptide loading onto MHC class I.

It has been shown that dimers can leave the ER to the *cis*-Golgi from where they cycle back to the ER [2]. Thus, another step of QC at the *cis* side of the Golgi apparatus is needed to hinder their egress to the cell surface and ultimately direct their route back to the ER. This retrograde pathway is highly mediated by calreticulin [3]. During the ER-Golgi cycle, the PLC is believed to assist the heavy chain- β_2_m dimers to bind specific peptides into their binding groove [4]. Susbequently, the peptide-HC-β_2_m complexes dissociate from the PLC and move as completely folded molecules through the secretory pathway to the cell surface to elicit an immune reponse by cytotoxic T cells (CTL) in case of infection.

Controlling the post-ER trafficking of MHC class I and their ER-Golgi cycle is very vital to the health of the cell. Any uncontrolled trafficking might lead to the escape of empty dimers or FHC to the cell surface contributing to certain diseases, such as spondyloarthropathy [5, 6]. Furthermore, empty dimers or monomers of MHC class I at the cell surface can bind peptides [7, 8] and become recognized by CTL. Thus, they can trigger the death of a healthy cell by binding to exogenous non-self peptides from the extracellular space.

Despite the flood of scientific studies that exist in the literature on the post-ER QC of partially folded MHC class I, there is no single mechanism underlying the behavior of different MHC class I allotypes during the ER-Golgi cycle. For instance, it is well reported that partially loaded human allotypes, HLA-B5 and HLA-A2, can reach the Golgi where they undergo peptide-loading and exit to the cell surface [9], whereas dimers of HLA-B27 accumulate in a subcompartment of the ER where they are destined for degradation by ubiquitination [10]. Additionally, murine H-2Kd get sorted at the cis-Golgi cisternae [11], and can reach the cell surface even in a dimeric form. However, dimers or FHC of H2-Kb cannot make it to the cell surface [12, 13] though dimers- but not FHC- can reach post-ER compartment from where their trajectory either back to the ER, or to the cell surface, or to degradation is not fully discerned.

In this study, we employ the *in vitro* rapamycin-trapping assay to further investigate the destiny of H-2Kb trafficking from a post-ER compartment. The assay depends on the generation of recombinant proteins tagged to FK506 binding protein (FKBP) or FKBP-rapamycin binding protein (FRB) that can interact upon addition of rapamycin. Our results show that recombinant H-2K^b^ mimic the localization pattern of endogenous molecules, namely they are capable of exiting the ER to the ER- Golgi intermediate complex (ERGIC) and/or cis-Golgi compartments. Furthermore, recombinant H-2Kb fused with FKBP or FRB are able to cycle back to the ER as revealed by their trapping with an ER localized marker. Trapping of H-2Kb was also exhibited at the cell surface, demonstrating possible sorting of partially folded molecules from the Golgi to the cell surface. Our results demonstrate the plausible usage of the rapamycin assay to assess the trafficking of proteins especially in diseases and under therapeutic studies. Finally, it paves the way to further explore the peptide-rescue function of unloaded MHC class I in post-ER compartments through their interaction with certain members of the PLC complex.

## Methods

### Mammalian cells and transfections

Mouse embryonic fibroblasts (MEF), untransfected or stably expressing H-2K^b^-GFP, were obtained from Michael Edidin (Johns Hopkins University, Maryland, US). Cells were cultured in DMEM and supplemented with 10 % heat-inactivated FCS and PSG (100 IU/ml penicillin, 100 mg/ml streptomycin, and 2 mM glutamine). Plain cells were transiently transfected using the FuGENE reagent (Roche Molecular Biochemicals, Indianapolis, IN). For single transfection, 50 μl OptiMEM were mixed with 2 μl FuGENE and 400 ng/μl DNA. For multiple transfection, 50 μl OptiMEM were mixed with 3 μl FuGENE and 200 ng/μl of each DNA. The OptiMEM/FuGENE/DNA mixture was kept at room temperature for 30 min and then added to cells that were already in 120 μl of fresh media. For the transfection to occur, the cells were incubated for 4 to 5 h with the FuGENE mixture. Afterwards, 400 μl of new media were added, and cells were incubated at 37 °C with 5 % CO_2_ overnight. The cell line, 293 T (ATCC® CRL-3216), is a highly transfectable derivative of human embryonic kidney 293 cells, and contains the SV40 T-antigen.

### Electroporation of Jurkat cells

One million cells were centrifuged at 200 x g for 10 min at room temperature (RT). 2 μg of DNA were mixed in 82 μl Nucleofector solution V (Amaxa) and 18 μl of supplement solution 1 at RT. In addition, the Nucleofector and supplement solutions were only mixed in the quantities required for each experiment to make sure that freshly mixed solutions were always used. After centrifugation as above, the supernatant was discarded, and the pellet was resuspended in 100 μl of the DNA/solution mixture. The mixture was then transferred to a sterile Amaxa cuvette, and cells were transfected using program X-001. The cells were then left at RT for 10 min. Meanwhile, RPMI medium (1.5 ml) was equilibrated in the incubator to 37 °C. 500 μl of the equilibrated medium was then added into the cuvette (very smoothly the cells were drawn into and out of the pipette tip three times, such that all sedimented cells were resuspended). The transfected cells were then plated and transferred immediately to the incubator at 37 °C with 5 % CO2. After 24 h, 1 ml of the supernatant was discarded, again care was taken not to disturb the sedimented cells. The remaining 500 μl cells were resuspended and half of the amount was used for the first-day experiment. To the remaining cells, 1250 μl of fresh media was added, and they were left to grow for another 24 h for the second-day experiment. For the first-day experiment, 250 μl of (about 600,000 cells) were splitted into three different wells 83 μl/each of a 6-well Ibidi chamber (Ibidi, Munich, Germany). The wells of the chamber were coated with 0.1 % polylysine. L-polylysine (Sigma) for 40 min at RT, the wells were then washed with water, and dried in the incubator for 10 min. Higher density of cells was bathed in a lower volume of buffer to keep them in the field of vision. Then to each of the wells, we added 117 μl of CO_2_-independent buffer. Three different experiments were performed in parallel.

### Antibodies

The following monoclonal antibodies (mAbs) were used: rabbit anti-GM130 (against the cytosolic tail of GM130 and marking mostly the *cis*-Golgi cisternae, purchased from BD Biosciences); rabbit anti-p58 (against p58 proteins in the ERGIC, a gift from Dr. Jaakko Saraste, Bergen, Norway); rabbit anti-PDI (against PDI proteins in the ER, a gift from Dr. Irina Majoul, Lübeck University, Germany); anti-P8 serum (against H-2Kb, a generous gift from Sjaak Neefjes, Netherlands Cancer Institute, Amsterdam), rabbit anti-EEA1 (as an early endosomal marker, BD Laboratories), α6F monoclonal antibody against Na-K-ATPase α1-subunit (α6F) [14], and the rabbit anti-murine tapasin antibody, 2668, against residues 11 to 34 [15].

### Chemicals and peptides

The peptide SIINFEKL (SL8) (from ovalbumin, 257–264; purified by HPLC) was purchased from Biosyntan (Berlin, Germany), dissolved with a stock concentration of 2.5 mM, and used at a final concentration of 10 μM. Peptides were added to the cells, without electroporation, for one to two hours.

Cycloheximide (purchased from Applichem; lot 9 V001009) is used as an inhibitor of protein biosynthesis in eukaryotes by blocking translational elongation. was mixed in water to a stock concentration of 10 mg/ml. The percentage of viability of cells as tested by trypan blue was about 80 % in the presence of 50 μg/ml of cycloheximide. Thus, we used this concentration throughout all the conducted experiments. Rapamycin (Sigma; lot 039 K4015) was mixed in DMSO at a stock concentration of 1 mM and used at 1 μM. Rapamycin is a small chemical with several roles among which it is believed to mediate the interaction of FKBP and FRB domains through its interface.

To avoid any effects of rapamycin on the trapping of freshly synthesized molecules, cells were pretreated with cycloheximide for ten minutes prior to rapamycin addition.

### Isolation of DNA

Plasmid preparations from small overnight cultures (1–5 ml) were either obtained using the QIAprep spin kit according to supplier’s protocol (Qiagen, Hilden, Germany), especially if the DNA was used for sequencing, or prepared using the rapid boiling miniprep method [16].

### Immunofluorescence and temperature block

To arrest trafficking of class I molecules in the ERGIC, cells expressing green fluorescent protein (GFP)- H-2K^b^ fusion proteins were incubated for 2 h at 15 °C and then fixed for 15 min at 15 °C with 4 % paraformaldehyde in phosphate-buffered saline (PBS) [17]. To accumulate class I molecules in the Golgi apparatus, cells were incubated for 2 h at 20 °C [18] and then fixed for 15 min at 20 °C with 4 % paraformaldehyde in PBS. After fixation, cells were permeabilized and stained with the indicated antibodies for one hour at room temperature. To detect H-2K^b^ cycling, cells were cotransfected with Ii and H-2K^b^ prior to their treatment with cycloheximide and rapamycin. Ii corresponds to the invariant chain associated with MHC class II moleucles and is usually used as an ER marker. Cells were then fixed and observed using a Zeiss LSM 510 confocal microscope. Cells that were cotransfected with FKBP or FRB constructs were grown in the chambers of plastic microscope slides (Ibidi, Martinsried, Germany) and fixed with paraformaldehyde without permeabilization in the wells. The cells were then observed directly in the chambers without any mounting using confocal microscope, LSM 510.

### Pearson’s coefficient

For quantification, the plugin JACoP version 2.0 was used in imageJ [19]. The pearson’s coefficient of the intensities of the two channels was plotted by the red channel on the x-coordinate (organelle markers) and the green channel on the y-coordinate (H2-Kb). Pearson’s coefficient provides an approximate of the rate of linear association between the two used fluorochromes. Its values range between −1 and 1 with zero no correlation and negative values for negative correlation. Region of interest (ROI) from selected cells was cropped, then channels were split, and quantified using JACoP plugin.

### COPII vesicle formation assay

The COPII generation assay was carried out as described by [3, 20, 21]. Briefly, mouse fibroblasts were radiolabelled with [^35^S]-methionine for 30 min and then harvested, resuspended in a low osmolarity buffer, and broken with 40 passes through a 22G syringe needle. After sedimenting nuclei and unbroken cells, the medium-speed pellet (15,000xg, 5 min) was washed twice and used as donor membranes for microsomes. Budding reactions consisted of microsomal membranes, pig brain cytosol, ATP regenerating system, and 0.2 mM GTP. Vesicles were isolated from the supernatant resulting from a 15,000 x g spin and sedimented by a 100 000 x g centrifugation, lysed in 1 % digitonin in 50 mM Tris-Cl (pH 7.5) and 150 mM NaCl, and radiolabelled proteins were then immunoprecipitated using rabbit serum specific for the GFP tag on H-2K^b^ (Fig. 4, lower panel), or as a control for budding with Na^+^/K^+^-ATPase antibodies (Fig. 4, upper panel). A peptide specific for H-2K^b^ (sequence SIINFEKL in the single letter amino acid code) was added to vesicles and to microsomes during lysis at 1 μM final concentration. Samples were treated with EndoF1 before SDS–PAGE, except for one sample that was used as a size reference band for class I heavy chain. Bands were quantified using ImageJ (1.44p) analysis tool an their relative densities were plotted using the highest peak of the budding reaction, donor membranes with peptides, as a standard.

### Immunoprecipitation

293 t cells were transfected with Venus-FRB- K^b^ or cotransfected with Venus-FRB-tapasin and Venus-FRB-K^b^. At 6 h post transfection, cells were labelled for 14 h with ^35^S-Met/Cys. Cells were lysed using 1 % digitonin. IP was perfomed with anti-H-2K^b^ (P8).

## Results and discussion

### Endogenous and recombinant H-2K^b^ molecules accumulate in a post-ER compartment

**Fig. 1:**
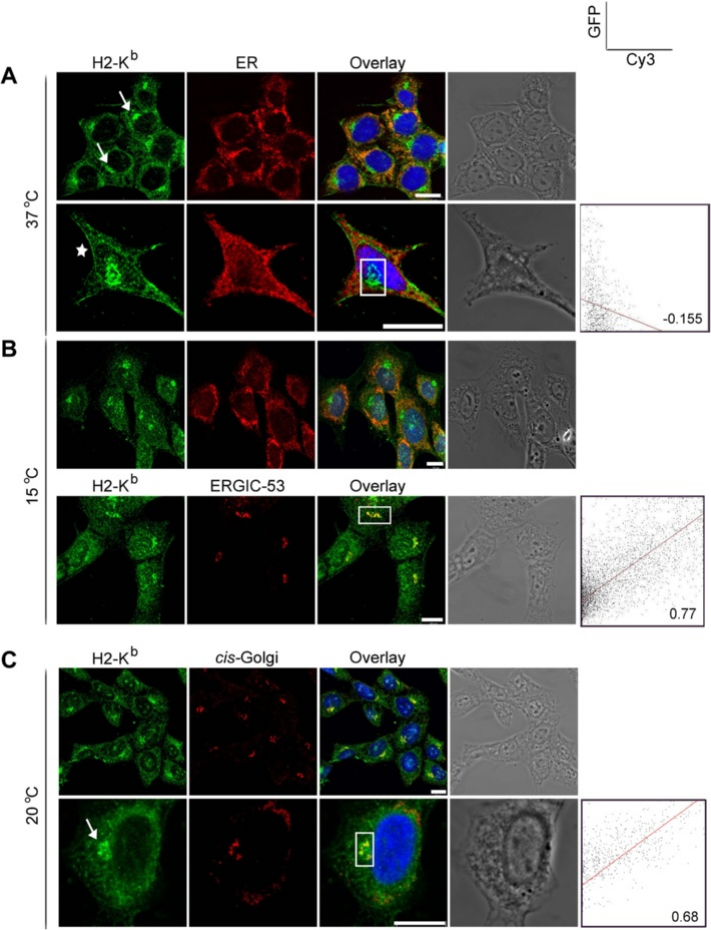
Endogenous H-2K^b^ accumulate in a juxtanuclear region and can reach the cell surface. MEF cells were fixed, permeabilized, and double stained for H-2K^b^ with anti-P8 serum and for one of the early secretory organelles, either ER (anti-PDI), ERGIC (anti-p58), or cis-Golgi (GM130). Prior to fixation, cells were either kept at 37 °C or incubated for 2 h at 15 °C or 20 °C, panels **b** and **c**, respectively. Most of the cells showed, in addition to the ER pattern, a juxtanuclear accumulation (**a**, arrows) that partially overlapped with the ERGIC and *cis*-Golgi stains. Some of the cells also showed weak cell surface stain (asterisk). Nuclei were stained with Draq5 as depicted in blue. All samples are shown in a bright-field mode to delineate the overall structure of the stained cells. Region of interest (ROI) from selected cells is quantified and a scatter plot with pearson’s coefficient is given.Scale bars, 10 μm

To examine whether the localization of H-2K^b^ is similar to previously reported data, we stained MEF cells with anti-P8, an antiserum against the cytosolic tail of H-2K^b^ [22]. Endogenous H-2K^b^ showed an ER-like stain in addition to a juxtanuclear accumulation (Fig. 1a; arrows). This accumulation resembled the ERGIC and *cis*-Golgi markers (Fig. 1b, c). However, even blocking the exit of H-2K^b^ from these compartments by low-temperature incubations (15 °C for the ERGIC, and 20 °C for the Golgi [23]), the pearsons’ coefficients for both the ERGIC and cis-Golgi channels with respect to H-2K^b^ channel did not show very high correlation with values ranging between 0.68 and 0.77, respectively. The cell surface was also stained, though weakly, in some cells (asterisks). Similar to previously reported data, endogenous H-2K^b^ exit the ER as partially or fully-loaded and reach post-ER compartments, which cannot be discerned by simple temperature blocks and colocalization comparisons. In addition, a small pool of the molecules can reach the cell surface.

**Fig. 2:**
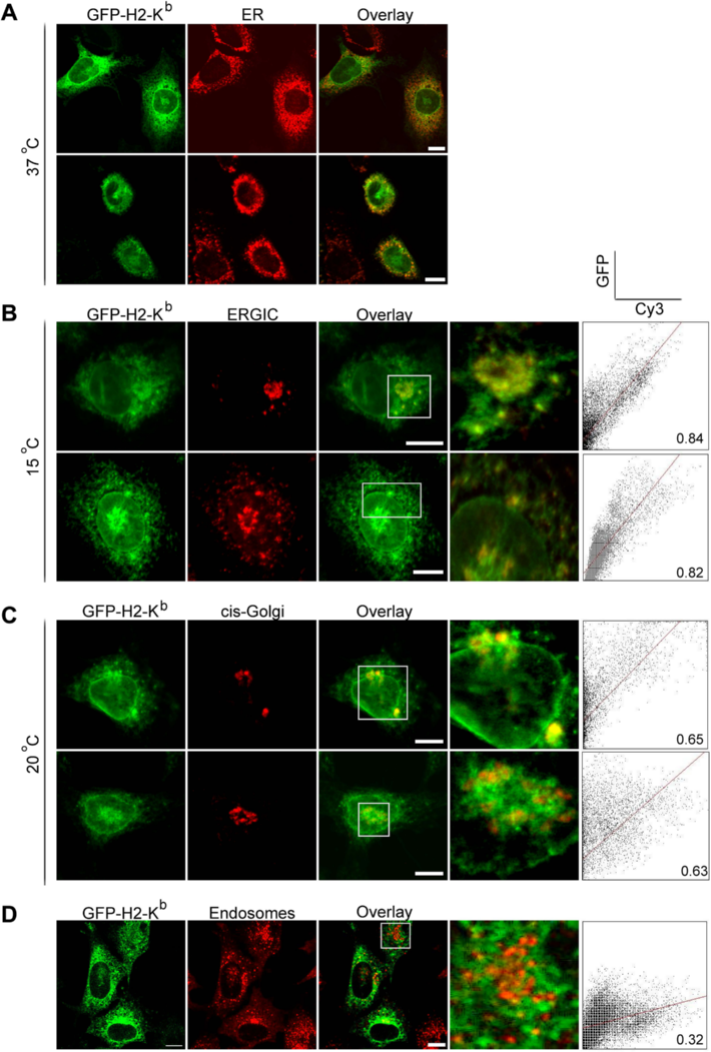
Recombinant GFP-K^b^ also form a post-ER accumulation. MEF cells transiently transfected with GFP-K^b^ were fixed, permeabilized, and stained with antibodies against PDI (**a**), p58 (**b**), or GM130 (**c**). Prior to fixation, cells were either kept at 37 °C or incubated for 1.5 h at 15 °C (**b**) or 20 °C (**c**). Scatter plots with pearson’s coefficient depict the fluorescence intensities of the secondary Ab (Cy3) on the x-axis and the GFP fluorescence on the y-axis. Scale bars, 10 μm

We next compared the endogenous expression pattern with the green fluorescent protein (GFP) fusion of H-2K^b^, GFP-H-2K^b^, in which the ER-lumenal amino terminus of H-2K^b^ is fused to the carboxy terminus of GFP [2]. We used an N-terminal fusion, rather than the C-terminal fusion used by Edidin and coworkers [24]. MEF cells expressing recombinant H-2K^b^-GFP were stained with different organelle markers. Then the GFP fluorescence was compared with that of the organelle antibody stain. In Fig. 2a, transfected cells were stained with anti-PDI (protein disulfide isomerase), an antibody that labels the ER peripheral tubules. GFP-K^b^ fluorescence was found in the central rough ER sheets, including the nuclear envelope, rather than the ER peripheral tubules. Upon 15 °C block, again inhibiting exit from the ERGIC, about 30 % of the cells showed a complete overlap of the accumulated molecules with the ERGIC area stained with anti-p58 antibody (Pearson’s coefficient ~ 0.83), and about additional 10 % of the cells showed a partial overlap (Data not shown) (Fig. 2b). A similar distribution pattern was observed when the accumulation of GFP-K^b^ was compared with the *cis*-Golgi marker, GM130, at 20 °C (Pearson’s coefficient ~ 0.64; Fig. 2c). The post-ER accumulation was not the result of endocytosed molecules from the cell surface, since there was no overlap between the fluorescence of GFP-K^b^ and the endosomal marker, EEA1 (Pearson’s coefficient ~ 0.32; Fig. 2d). Both H-2K^b^ endogenous and recombinant H-2K^b^-GFP molecules exhibit similar intracellular distribution though the cell surface signal was not detected with the GFP fusions. The intensity signal at the cell surface could be low due to the high expression level of H-2K^b^ in the transfected cells. Thus, the ratio of partially to fully loaded molecules is highly masking the presence- if any- of cell surface proteins.

Taken together, our results so far suggest that the trafficking of a large pool of both endogenous and recombinant H-2K^b^ is jammed outside the ER. The calculated pearsons’ coefficients did not show complete correlation between the colocalized H-2Kb and organelle channels, but was slightly higher for the ERGIC than the cis-Golgi. This underlying fact points up the possibility of the involvement of another quality control compartment that might resemble as well the ERGIC and cis-Golgi compartments. This behavioral phenotype compounded with the cell surface expression of molecules might be restricted to MEF cells and is prone to variation based on the origin and overall characteristics of the tested cell line.

### H-2K^b^ exit the ER in COPII vesicles

**Fig. 3:**
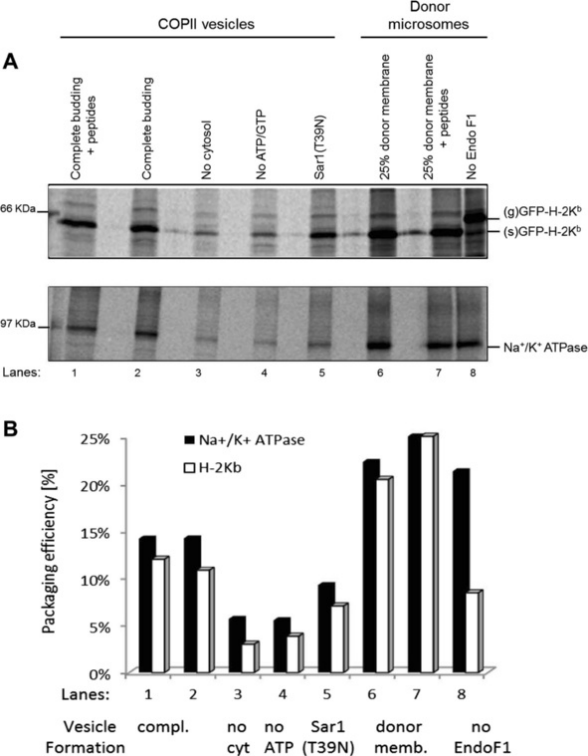
H-2K^b^–GFP molecules exit the ER in COPII vesicles. COPII vesicles were generated by an *in vitro* reaction from MEF cells. Controls for this reaction were the omission of cytosol (lane 3), ATP (lane 4), or addition of dominant-negative Sar1 (T39N; lane 5). COPII vesicles or the corresponding donor microsomal membranes (after the reaction; lane 6–8) were lysed with detergent (in the presence of 10 μM peptide as indicated in lanes 1 and 7). H-2K^b^–GFP (upper panel) and Na^+^/K^+^ATPase (lower panel) were sequentially immunoprecipitated from the lysates with anti-GFP serum and α6F antibodies, respectively as depicted in panel **a**. Immunoprecipitates were treated with EndoF1 (lanes 1–7). The band intensities were quantified and plotted in panel (**b**). One out of three experiments is shown here

To characterize the nature of the trafficking pathway that H-2K^b^ follow to exit the ER, COPII vesicles that carry proteins from the ER to the ERGIC in mammalian cells were targeted [25]. COPII vesicles cannot be isolated from extracts due to their short lifetime in cells. Thus, they were produced from microsomal membranes by an *in vitro* budding reaction that Springer *et al.* group has established. [2, 26]. Different COPII formation reactions were performed with microsomes of MEF cells in the presence of cytosol (as a source of COPII components), ATP, and wild type recombinant Sar1 protein (the GTPase that drives COPII budding). Budded vesicles were then isolated by differential centrifugation, lysed, and GFP-H-2K^b^ or Na+/K^+^ ATPase, as a control protein for trafficking [3], were immunoprecipitated with anti-GFP serum and anti-Na^+^/K^+^ ATPase antibodies, respectively (Fig. 3a). Proteins were then detected by SDS-PAGE and autoradiography. In the absence of cytosol (lane 3), absence of ATP (lane 4), or in the presence of mutant Sar1(T39N) as a specific inhibitor for COPII vesicles (lane 5), the amounts of H-2K^b^ and ATPase found in the vesicle fraction were reduced (Fig. 3, lanes 3 to 5). The packaging efficiency for all the lanes was quantified relative to lane 7 (Fig. 3b). Comparable budding efficiency for both proteins was measured, with four fold reduction in lanes 3 and 4. This indicates that the isolated vesicles were indeed COPII vesicles. In lane 8, sample was left untreated with EndoF1 revealing a shift of H-2Kb alleles from sensitive form (s)GFP- H-2Kb to glycosylated form (g) GFP- H-2Kb. Thus, H-2Kb in COPII vesicles are EndoF1-sensitive, revealing their trafficking out of the ER en route to the ERGIC and cis-Golgi but they can not travel beyond the median Golgi. Interestingly, addition of peptides to the complete budding reaction (lane 1) or to donor membranes (lane 7) did not increase the intensity of packaged proteins pointing to the existence of mostly unfolded heavy chains H-2Kb in COPII vesicles. This can also point to the ER-egress of not only partially folded proteins, but also heavy chains of H-2K^b^ molecules.

### *In vitro* FKBP/FRB rapamycin-trapping assay is adopted to further confirm the trafficking of H-2K^b^

To follow up on the above observation we used a rapamycin trapping assay, which utilizes the non-covalent crosslink that occurs between two domains, FKBP (FK506 binding protein) and FRB (FKBP-rapamycin binding domain), upon addition of rapamycin [27]. These domains were conjugated to the C-termini of the Venus and Cerulean fluorescent proteins [28]. Cerulean- FKBP and Venus-FRB were then fused to the amino termini of H-2K^b^, tapasin, or various organelle markers (Table 1); in this way, triple fusions, such as Venus-FRB-K^b^ and Venus-FRB-tapasin were generated.

**Table 1:** DNA constructs

**Name** ** (restriction enzyme)**	**Sequence**	**Amplification purpose**
EGh_FRB- H-2K^b^_fwd (HindIII)	5’- cccAAGCTTcgggcccacactcgctg	H-2K^b^ in ss-EGFP- FRB
EGh_FRB- H-2K^b^_rev (BamHI)	5’- gtGGATCCtcacgctagagaatgagg	H-2K^b^ in ss-EGFP- FRB
EGh_FRB-tpn_fwd (HindIII)	5’-cccAAGCTTcgggaccagaggcgatcg	Murine tapasin in ss-EGFP-FRB
EGh_FRB-tpn_rev (BamHI)	5’- gtGGATCCttactgtgacttctttgagt	Murine tapasin in ss-EGFP-FRB

EGFP-H-2K^b^ was generated as described in Garstka et al. 2007. The Cerulean-FKBP-Ii and Cerulean-FKBP-GPI constructs were obtained from Dr. Jennifer Lippincott-Schwartz (NIH, Bethesda, US). The other constructs, Cerulean-FKBP-p58, Venus-FRB-K^b^, and Venus-FRB-Tapasin were generated using the following primers

**Fig. 4:**
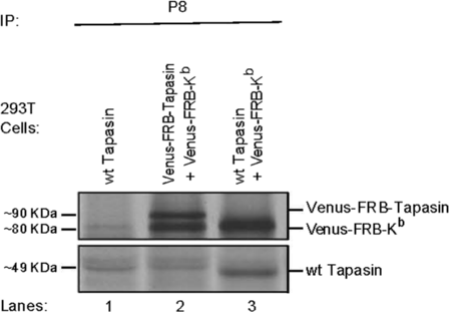
Examining the proper size of recombinant Venus-FRB-K^b^ molecules in 293 T cells. Venus-FRB-K^b^ showed an estimate size of 80KDa and associated with both Venus-FRB-Tapasin (lane 2) and wild type tapasin (lane 3)

Initially, we examined the proper size of these constructs in 293 T cells (Fig. 4). 293 T cells were either left untranfected (lane 1) or cotransfected with Venus-FRB-K^b^ and Venus-FRB-tapasin (lane 2) or with Venus-FRB-K^b^ and wt Tapasin (lane 3). H-2K^b^ were pulled down with anti-P8 antibody (lanes 1 to 3) followed by blotting with anti-tapasin antibody, #2668. Venus-FRB-K^b^ had the expected size of approximately 80 kDa and interacted with both Venus-FRB-Tapasin (lane 2) and wild type tapasin (lane 3).

**Fig. 5:**
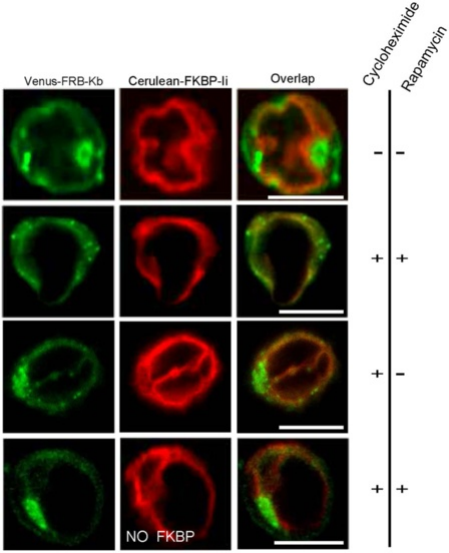
Cycling of Venus-FRB-Kb from post-ER compartment back to the ER. Venus-FRB-Kb is trapped in the ER by Cerulean-FKBP-Ii upon addition of rapaymcin. The fluorescence intensity of Venus-FRB-Kb accumulation from the first panel disappears upon the addition of rapamycin (1 mM) for 2 h (second panel). Trapping of Venus-FRB-Kb molecules does not occur in the controls where rapamycin is absent (first and third panels) or in the presence of Cerulean-Iip35 that lacks the FKBP domain (fourth panel). Scale bars, 10 μm

Because of the previous observation by us and others that class I molecules cycle between the ER and the Golgi at steady state [11, 20], we next examined the cycling of accumulated MHC class I molecules in Jurkat cells using the rapamycin trapping assay. MEF cells were substituted by Jurkats as the latter was easier to double and triple transfect. In addition, Jurkats are lymphocytes with a better system that mimics and coordinates the normal behavioral function of MHC class I and the peptide loading complex proteins. Lastly, Jurkats possess a smaller surface area, which facilitates the tracking of recycled molecules. We cotransfected Jurkat cells with Venus-FRB-K^b^ and Cerulean-FKBP-Ii. Cells showed Cerulean-FKBP-Ii expression in the ER, whereas Venus-FRB-K^b^ was detected not only in the ER, but also at the cell surface and in intracellular accumulations (Fig. 5, first panel). If Venus-FRB-K^b^ cycles back to the Golgi from post-ER compartment, then accumulating molecules should be trapped by Cerulean-FKBP-Ii in the ER upon addition of rapamycin. Since, Ii protein is permanently residing in the ER, then we expect any recycling H-2Kb to be trapped by Ii resulting in a decrease of the fluorescence intensity of the post-ER accumulated molecules over time. To rule out the artifact of Ii trapping newly synthesized Kb in the ER, we incubated the cells with cycloheximide for ten minutes prior to rapamycin addition. Cycloheximide inhibits the elongation of protein translation and thus, allows us to evaluate the ER-trapping of accumulated H-2Kb rather than Kb already present in the ER. Consistent with our hypothesis, we observed that the accumulation of Venus-FRB-K^b^ disappeared after one hour treatment with rapamycin (Fig. 5, second panel). In the controls, without adding rapamycin or in the absence of an FKBP domain, the accumulated pool of H-2Kb persisted even at prolonged incubation period. This trimmed configuration of Cerulean-Ii, “No FKBP”, offers a good control to directly assess the recycling and trapping of Venus-FRB-Kb molecules. It is worth noting that the around 40 % of the cells did not show a disintegration of the accumulated molecules.

### The disappearance of accumulated Venus-FRB-K^b^ over time is not due to the disruption of the Golgi apparatus

**Fig. 6:**
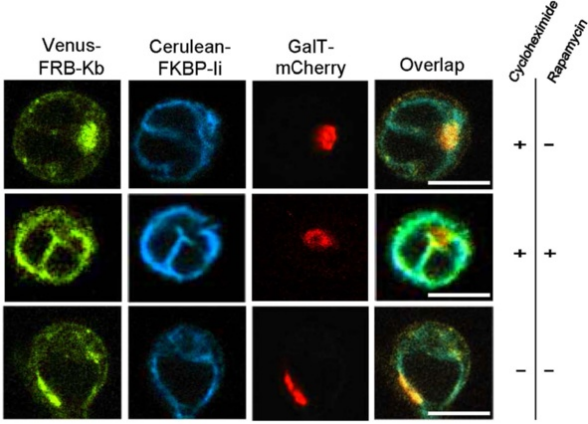
Integrity of the Golgi apparatus in the presence of rapamycin. The Golgi structure is maintained even after six hours incubation of Jurkat cells with cycloheximide and rapamycin. Jurkat cells triple transfected with Venus-FRB-Kb Cerulean-FKBP-Ii, and GalT-mCherry show intact Golgi even after six hours treatment with cycloheximide and rapamycin. Scale bars, 10 μm

Treatment of cells for prolonged period with cycloheximide might disrupt the structure of post-ER organelles, since they depend on newly synthesized proteins to maintain their integrity. On a related note, the trafficking of Scy11 between the Golgi and ER is required for the maintenance of the Golgi apparatus [29]. To test whether the relocation of Venus-FRB-K^b^ molecules to the ER is happening as a result of its trapping and not due to the Golgi disassembly, we triple transfected Jurkat cells with Venus-FRB-K^b^, Cerulean-FKBP-Ii, and GalT-mCherry. GalT (1,4-galactosyltransferase) is a marker for the trans-Golgi membranes and the trans-Golgi network. Cells were then either treated with cycloheximide in the presence or absence of rapamycin, or left untreated. The Golgi structure (as detected by the fluorescence of GalT-mCherry) was maintained even after prolonged (6 h) incubation with cycloheximide (Fig. 6, first panel). Thus, the trapping effect is not mediated by the disintegration of the Golgi structure, but rather reflects the recycling of our tracked proteins. Consistent with our hypothesis, MHC class I trapping by Cerulean-FKBP-Ii was again observed (Fig. 6, second panel). This confirms the cycling of Venus-FRB-K^b^ molecules between the *cis*-Golgi and the ER and validates our assay.

### Live- cell imaging to examine the cycling of recombinant Venus-FRB-K^b^

**Fig. 7:**
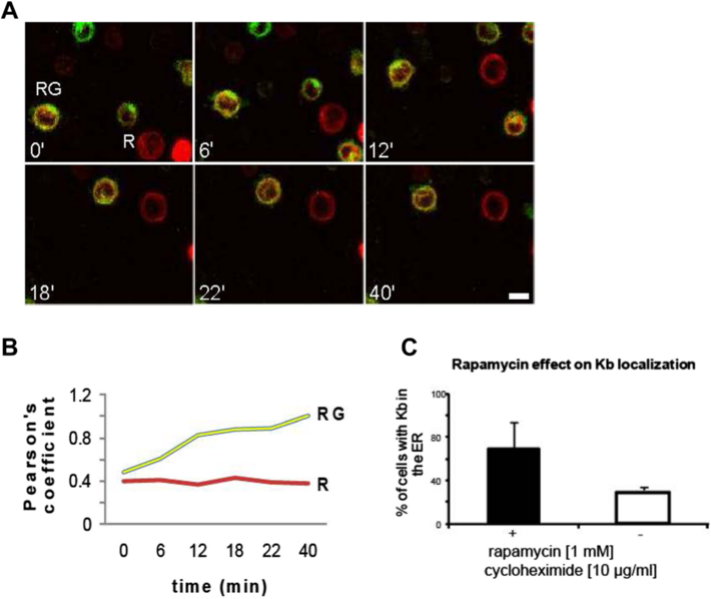
Live cell imaging for Venus-FRB-Kb cycling. (**a**) Six different snapshots of live Jurkat cells coexpressing Venus-FRB-Kb and Cerulean-FKBP-Ii (double-transfected red and green cell = RG) or expressing only one of the constructs (only Ii in red = R cell) were taken from a 45 min movie (images were captured at a 2-min interval; snapshots are revealed at different allocated time points). Each image consists of four compiled z-stacks, where each ∆z = 1 μm. Before recording the movie, cells were transferred to a 37 °C heat stage in CO_2_-independent buffer and incubated first with cycloheximide (10 μg/ml) for 10 min and then with rapamycin (1 μM). Pearson’s coefficient was calculated from each image by defining a circular region of interest (ROI) around the Golgi area where the green fluorescence intensity of Venus-FRB-Kb accumulates (an arrow pointing to the circle in the first panel). For the cell only expressing Cerulean-FKBP-Ii in red (R cell), the ROI was drawn around the entire cell. (**b**) The graph shows the time-dependent change in Pearson’s coefficient based on the variation of the green fluorescence intensity with respect to the red fluorescence in the chosen ROI. There is an increase in Pearson’s coefficient for the RG but not for the control (R) cell. (**c**) There is a 25 % increase in the percentage of cells that show Venus-FRB-Kb localization in the ER upon addition of rapamycin (*p < 0.02). The error bars correspond to the SEM values from 2 sets of experiments (*n* > 250)

We next followed the fluorescence intensity of accumulated Venus-FRB-K^b^ molecules in live cell microscopy over a time period of 45 min from the time of rapamycin addition, which is ten to fifteen minutes after addition of cycloheximide. The movie consists of concatenated images taken at time interval of 2 min. Each image is in turn obtained by a compilation of four z-stacks (z = 1 μm). Six different images taken from the movie at time points: 0, 6, 12, 18, 22, and 40 min are depicted in Fig. **7**. The fluorescence intensity (FI) of Venus-FRB-K^b^ was analyzed by calculating Pearson’s coefficient from a circular region of interest (ROI) drawn around the region in the double transfected cell (red and green = RG), where the green intensity is more concentrated and denser than the red intensity (RG cell in Fig. 7a). As a control, Pearson’s coefficient was calculated from an ROI around a single transfected cell expressing only Cerulean-FKBP-Ii (R cell in Fig. 7). The time-dependent decrease of the accumulated pool of the RG cell is demonstrated by the increase in the Pearson’s coefficient values over time reaching almost 0.9 at around 40 min (Fig. 7b). For the control cell (R cell), Pearson’s coefficient was constant unaffected by rapamycin addition. Interestingly, the percentage of cells showing a morphology of MHC class I only in the ER, without any Golgi or cell surface expression, increased from 30 % to almost 70 % in the presence of rapamycin (Fig. 7c).

### Egress of H-2K^b^ to the cell surface upon addition of peptides

Finally we assessed the localization of Venus-FRB**-**K^b^ in MEF cells. Similar to endogenous H-2K^b^ and of GFP-H-2K^b^, they were mostly localized to the ER and showed an additional accumulation close to the nucleus with a weak delineation of the cell surface **(data not shown)**. Cells were pretreated with cycloheximide for 10 min- to avoid cross-linking of the nascent proteins in the ER- prior to their one-hour incubation with or without rapamycin.. As expected, after treatment with rapamycin, 60 % of the cells lacked a juxtanuclear accumulation and mostly showed an ER-like pattern for Venus-FRB**-**K^b^ with few vesicular structures that could be late endosomes **(data not shown)**. Trapping failed to occur in cells co-transfected with Cerulean-FKBP-Ii and Venus-Kb lacking the FRB domain (data not shown).

**Fig. 8:**
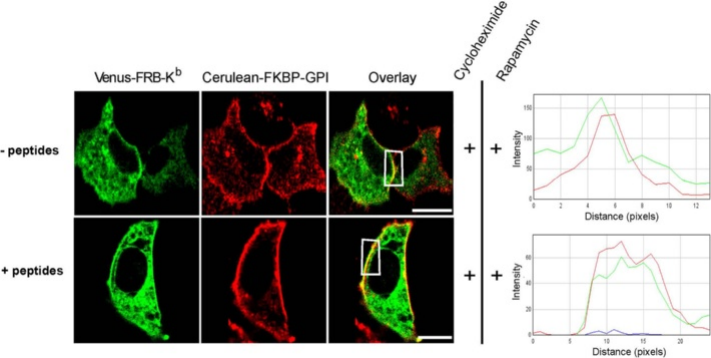
Preliminary data on the trafficking of H-2Kb from post-ER compartment directly to the cell surface. MEF cells were double transfected with Venus-FRB-K^b^ and Cerulean-FKBP-GPI. The invariant chain Ii is retained in the ER, whereas GPI can reach the cell surface. Cells treated with cycloheximide and rapamycin without addition of peptides showed Venus-FRB-K^b^ in the ER with a faint delineation of the cell surface. From the intensity RGB profile, the pseudo-color curves do not overlay. Cells treated with 10 μM of SIINFEKL peptides showed a clear cell surface stain that was more intense in the presence of rapamycin. The RGB profile depicts an overlay of the fluorophore curves at the same distance. Scale bars, 10 μm

We next tested whether the cell surface presence of Venus-FRB-K^b^ can be increased by trapping recombinant K^b^ at the cell surface by Cerulean-FKBP-GPI. The Cerulean-FKBP- protein is modified in the ER by its fusion with a GPI (glycosylphosphatidylinositol) peptide signal moiety that anchors it to the cholesterol-rich lipid microdomains of the plasma membrane and thus maintains its steady state localization at the cell surface [30]. For this, we double transfected cells with Venus**-**FRB-K^b^ and Cerulean-FKBP-GPI. To extend the residence time of Venus-FRB-K^b^ at the cell surface, we added 10 μM of high-affinity peptide (SIINFEKL in the single letter amino acid code) to the medium for one hour. As shown in Fig. 8, the addition of rapamycin to cells incubated with cycloheximide and peptides increased the fluorescence of Venus-FRB**-**K^b^ at the cell surface. This advocates the possible peptide-loading of H-2K^b^ in a post-ER compartment, rescuing their egress to the cell surface. More experiments should be done to reveal a clear statistics on the overall behavior of the cells.

## Conclusion

Several reports have examined proteins that undergo post-ER quality control; Examples include MHC class I partially-folded dimers that reach post-ER compartments from where they cycle back to the ER possibly by Bap31 [20] or calreticulin [3]; mutants of influenza hemagglutinin that leave the ER to reach the plasma membrane for degradation by endocytosis [31]; the N153D mutant of tissue non-specific alkaline phosphatase (TNSALP) [32], which leaves the ER and is retained in the cis-Golgi until degradation; and the ts405 mutant form of the vesicular stomatitis virus G protein (VSVG), which cycles between the Golgi and ER until it is finally degraded by ERAD [33].

In this work, we used an exquisite rapamycin-trapping assay to detect and confirm the trafficking and sorting of MHC class I molecules, H-2K^b^, from post-ER compartments.

We provide biochemical evidence that in MEF cells, H-2K^b^ exit the ER in COPII vesicles. This assay is well established and monitored earlier in Springer’s laboratory in Germany. The result provides independent biochemical evidence that indeed GFP-H2K^b^ can leave the ER, and that the sorting-QC that determines the intracellular transport of dimers is located in a post-ER compartment. Interestingly, GFP-Kb molecules were found in COPII vesicle fractions regardless of the presence of peptides.

In addition, we present microscopic evidence that the post-ER compartment where H-2Kb accumulate resembles closely the ERGIC and/or the cis-Golgi compartments. The central aim of this report was to assess the destiny of the trafficking of H-2Kb accumulated molecules. We were able to show that the majority of H-2K^b^ alleles can cycle back to the ER as revealed by the *in vitro and in vivo* trapping with an ER marker, Ii. Since not all the molecules showed similar cycling pattern, then we believe that there is a degradation pathway that releases the stress of accumulating immature proteins outside the ER. One possibility arising from the exit of unfolded proteins from the ER is their degradation in an ERGIC-expanded region [10] or in a QC compartment (QCC) [34]. However, given that the PLC assists the heavy chain- β_2_m dimers to bind peptides onto the class I peptide binding groove [4] and based on what we have shown that TAP is active in post-ER compartment, then there is a substantial possibility of loading partially-folded molecules with peptides during their ER-Golgi cycle, as a rescuing event to further open the gate for protein channeling to the cell surface.

The latter assumption is supported by our data on the trapping of H-2Kb at the cell surface by GPI. We showed that cells incubated with peptides exhibited a cell surface stain that was upregulated in the presence of rapamycin. Our report paves the way for further work to decipher the trafficking of H-2K^b^ to the cell surface and to unravel the chaperones or proteins that mediate H-2K^b^ trafficking and stability at the cell surface.

Finally, this assay can be extended to examine the cycling of other ER chaperones, such as tapasin, that has also been shown to partially exit the ER especially in lymphocytes.

## Footnotes

1 ^1^ Abbreviations used: **β**_**2**_**m**, beta-2 microglobulin; **BFA**, brefeldin A; **CTL**, cytotoxic T lymphocytes; **ER**, endoplasmic reticulum; **ERAD**, ER-associated degradation; **ER-QCC**, endoplasmic reticulum quality control compartment; **FHC**, free heavy chains; **FKBP**, FK506 binding protein; **FRB**, FKPB-rapamycin binding protein; **GFP**, green fluorescent protein; **GPI**, glycosylphosphatidylinositol; **HC**, (MHC class I molecule) heavy chain; **MHC**, Major histocompatibility complex; **PLC**, (MHC class I) peptide loading complex; **QC**, quality control.
